# Hard and Soft Tissue Augmentation with Single-Implant Restoration in the Esthetic Zone

**DOI:** 10.1155/2021/5737665

**Published:** 2021-11-17

**Authors:** Igor Ashurko, Nataliia Blagushina, Anisa Borodiy, Mary Magdalyanova

**Affiliations:** Department of Oral Surgery, E.V. Borovsky Institute of Dentistry of I.M. Sechenov First Moscow State Medical University (Sechenov University), Mozhaisky Val, 11 Moscow, Russia

## Abstract

The removal of the central incisor of the upper jaw is a challenging process, since the restoration of a single implant in the esthetic zone is of great responsibility. When tooth extraction with immediate implant placement is not possible, modern protocols imply the use of the socket preservation technique. This method of preserving socket properties significantly reduces changes that occur during the healing process of the postextracted area and along with soft tissue augmentation allows to achieve a satisfying aesthetic result. The aim of present case report is to assess capabilities of socket preservation concept and application of soft tissue augmentation in the anterior maxillary region. The treatment steps of a 35-year-old woman who needed to remove tooth 11 with further dental implant placement are described. The clinical case is of interest because after socket preservation and soft tissue augmentation, an acceptable aesthetic result was not reached. However, additional soft tissue augmentation made it possible to achieve the desired result, which was stable for 7 years.

## 1. Introduction

Rehabilitation in an aesthetic zone is often challenging. It is even more complicated when it is necessary to remove one of the teeth and replace it with an implant. There are many factors that influence peri-implant tissue stability in the anterior region. Therefore, several methods have been proposed to prevent bone loss and preserve the volume of soft tissue in the intervention area, including different timing protocols of implant placement and implant loading, specific implant positioning and developing a new implant design, the use of bone graft materials, and soft tissue augmentation procedures [1, 2]. After tooth extraction, the process of atrophy of bone tissue is triggered, which leads to a decrease in the volumetric parameters of the socket and a deterioration of the conditions to obtain a satisfactory aesthetic result of treatment [3]. One of the well-known methods to reduce the degree of alveolar bone loss is a socket preservation technique. The effectiveness of the method has been proven by many clinical and experimental studies [4, 5]. However, according to a number of authors, the use of the socket preservation alone does not fully compensate for the loss of volume of the alveolar ridge [6]. Many authors recommend soft tissue augmentation when working with implants in the anterior region [7–9]. It allows to create the most natural contour of the alveolar ridge and restore the lost aesthetics.

This article describes a clinical case of the removal of an inconsistent tooth with a technique to preserve the parameters of the socket, the subsequent implant installation with a two-stage soft tissue augmentation. This clinical case is interesting in that it not only confirms the need for the above manipulations to achieve a long-term functional and aesthetic result but also shows the problems the clinician may face.

## 2. Case Presentation

A 35-year-old female patient came to the clinic in order to change old metal-ceramic restorations on the upper and lower jaws. The patient was concerned about the appearance of her teeth. In particular, alignment of the gingival contours of the central incisors on the upper jaw was not sufficient.

According to the patient, the metal-ceramic constructions were made about 7 years ago. Prosthetic treatment was preceded by endodontic preparation of the teeth.

The patient is somatically healthy, and no chronic diseases or allergies have been reported.

### 2.1. Planning

During clinical examination, an asymmetry of the gingival contours of the frontal part of the upper jaw was revealed (apical displacement of the gingival margin of tooth 11).

Periodontal examination revealed a pocket of 12 mm in depth on the mesial side of the tooth root, 9 mm on the distal side, 6-8 mm on the palatal, and 4-5 mm on the vestibular sides.

**Figure 1:**
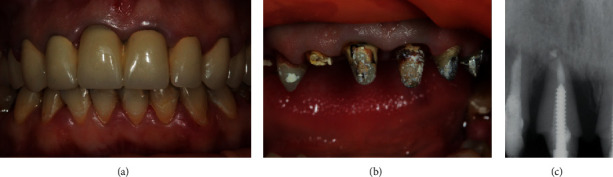
(a) Frontal area of the upper jaw. (b) After removal of metal-ceramic crowns. (c) Periapical X-ray of the tooth 11.

The intraoral periapical radiograph showed bone resorption in the apical direction in the form of a reduced bone density area in 2/3 of the radiolucent periapical lesion of the root length. An excess of root filling material was detected beyond the apex (Figure 1).

Since immediate implant placement was not possible in this case, we recommended extracting the tooth and performing the method of the socket preservation.

Following the main procedure, dental implantation, soft tissue augmentation, and subsequent prosthetics were planned to be performed.

### 2.2. Surgical Procedures

**Figure 2:**
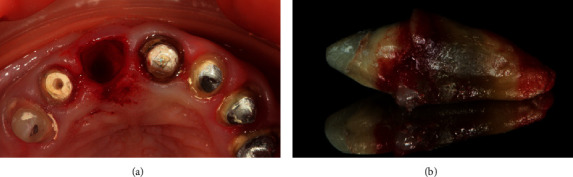
(a) Situation after tooth extraction. (b) Extracted tooth 11.

The patient received a prophylactic dose of antibiotic (1 g Amoxiclavi, LEK, d.d., Slovenia) 1 hour prior to surgery. Dissection of the circular ligament of tooth 11 was made using surgical blades 15c and 12d under local anaesthesia with 4% articaine hydrochloride (Ubistesin; 3M ESPE). Atraumatic extraction of the tooth was implemented (Figure 2). After curettage of the socket, it was rinsed with 0.05% chlorhexidine solution [10, 11].

**Figure 3:**
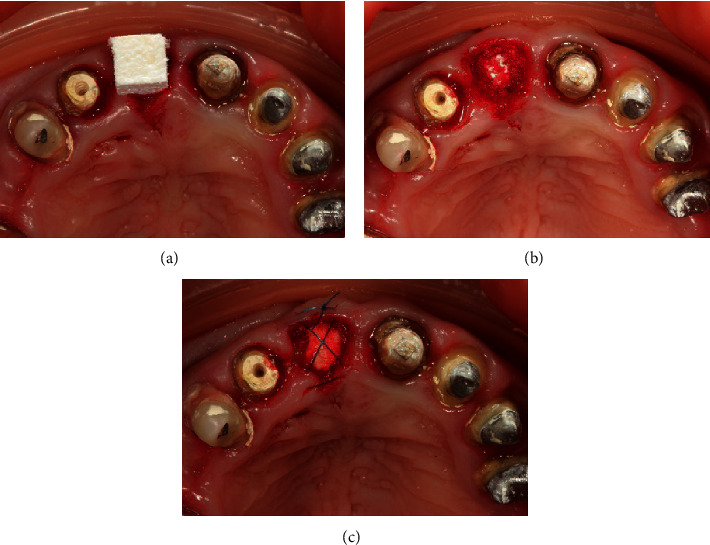
(a) Insertion of Bio-Oss collagen in a dry form. (b) The alveolus is completely packed with the material. (c) A resorbable membrane is laid, and stabilizing sutures are applied.

The socket was filled with Bio-Oss collagen bone substitute material (Geistlich Pharma AG). In the process of packing the material, it was gradually impregnated with blood. The bone-substitute material was covered with a Bio-Gide native resorbable collagen membrane (Geistlich Pharma AG), which was fixed by Prolene monofilament 6-0 cross suture (Ethicon W8005, Johnson & Johnson) (Figure 3).

For hermetic closure of the socket, we decided to install a temporary bridge construction over the defect.

Postoperative recommendations included rinsing with 0.2% chlorhexidine gluconate mouthwash solution (Corsodyl, GlaxoSmithKline) twice a day for a week and receiving antibiotic (1 g Amoxiclavi, LEK, d.d., Slovenia) twice a day for 5 days. To relieve pain, 100 mg of Nimesulide (Nise; Dr. Reddy's Laboratories Ltd., India) was prescribed. We also recommended to minimize trauma at the site; no special diet was proposed. The sutures were removed 14 days after surgical treatment.

**Figure 4:**
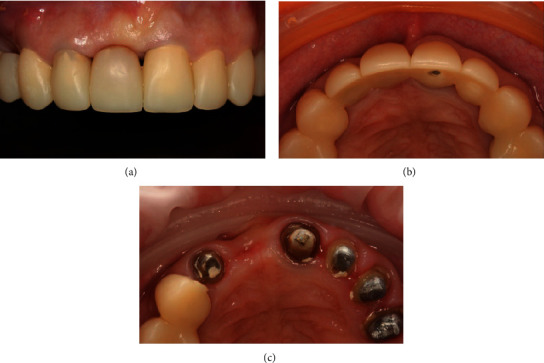
(a, b) 4 months after the operation. (c) After the removal of the temporary appliance, a change in the contour of the crest in the vestibular-oral direction.

The feasibility of implant placement was carried out at the 4 months postoperative visit. In the area of the previous surgical treatment, complete epithelialization of the socket was evident; the mucosa was pale pink, and no pathological changes were found. However, soft tissue deficiency was found in the area of the 11 tooth socket on the vestibular surface (Figure 4).

At the same time, on the basis of cone-beam computed tomography (CBCT), the adequate height and width of the alveolar ridge (bone volume parameters) for dental implant placement was determined.

The density of newly formed bone tissue in the area of previous socket augmentation corresponded to the normal density, without pathological changes. According to this clinical situation, we decided to place a dental implant with soft tissue augmentation using a free connective tissue graft (CTG) from the tuberosity area.

The patient received a prophylactic dose of antibiotic (1 g Amoxiclavi, LEK, d.d., Slovenia) 1 hour prior to surgery. Under local anaesthesia with 4% articaine hydrochloride (Ubistesin; 3M ESPE) using a 15c blade, the mucosa was dissected along the top of the alveolar ridge within teeth 21 and 12.

**Figure 5:**
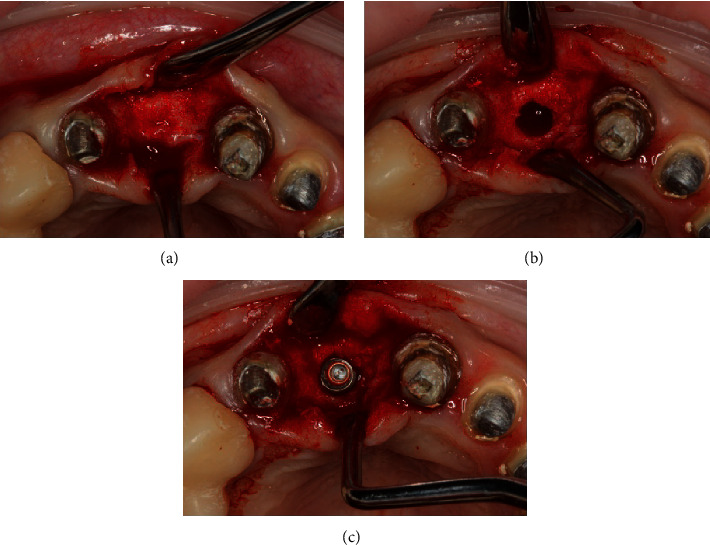
(a) The mucoperiosteal flap is detached. (b) The implant bed preparation. (c) The implant placement.

The mucoperiosteal flap was elevated. According to the following surgical protocol, an Astra Tech dental implant (Dentsply Sirona, York, Pennsylvania) 3.5 × 11 was placed at the site of the extracted tooth 11. The implant insertion torque was 35 N/cm; the implant stability quotient (ISQ) value was 60 (Figure 5).

**Figure 6:**
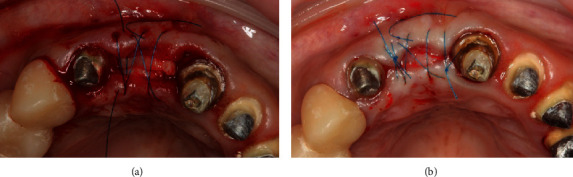
(a) Fixation of free connective tissue grafts from the maxillary tuberosity. (b) Final suturing.

Three fragments of CTG were taken from the maxilla tuber region on the right side and fixed to the vestibular flap with Prolene monofilament 6-0 horizontal mattress suture (Ethicon W8005, Johnson & Johnson) (Figure 6).

A temporary bridge construction was installed over the defect, which was modified taking into account the changes in the configuration of the alveolar ridge.

Postoperative therapy included rinsing with 0.2% chlorhexidine gluconate mouthwash solution (Corsodyl, GlaxoSmithKline) twice a day for a week and receiving antibiotic (1 g Amoxiclavi, LEK, d.d., Slovenia) twice a day for 5 days. To relieve pain, 100 mg of Nimesulide (Nise; Dr. Reddy's Laboratories Ltd., India) was prescribed. We advised to minimize trauma at the site; no special diet was recommended. The sutures were removed 14 days after surgical treatment.

**Figure 7:**
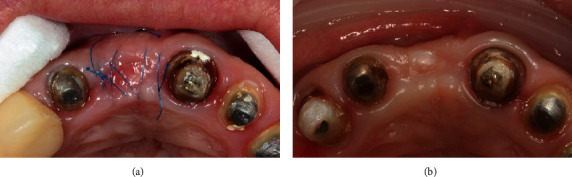
(a) Appearance of the alveolar crest 14 days after surgery. (b) 3 months after surgery.

Further follow-up evaluations occurred at 2 weeks. A sufficient volume of the alveolar ridge was visualized in the vestibulooral direction. However, at 3 months postsurgery, the soft tissue volume of the vestibular surface was not sufficient (Figure 7).

For a good aesthetic result, we made a decision about implant disclosure with one-stage soft tissue grafting procedure using deepithelialized free gingival graft.

**Figure 8:**
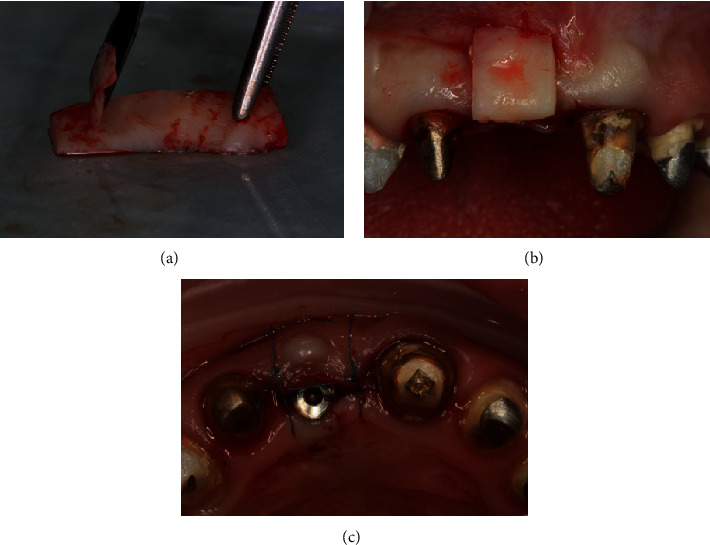
(a) Deepithelization of the free connective tissue graft from the hard palate. (b) Projection of the graft on the formed tunnel from the vestibular surface. (c) The graft is placed in the formed bed; the wound is sutured.

Under local anaesthesia with 4% articaine hydrochloride (Ubistesin; 3M ESPE) using a 15c blade, the mucosa was split along the top of the alveolar ridge within teeth 21 and 12. Splitted flap was stripped away; screw end cap was removed. The tunnel was performed on the vestibular site. Free gingival graft with a size of 12 × 6 *мм* and thickness of 2 mm was harvested from the palate area on the right side. The graft was deepithelialized using an 11с blade. Then, it was folded in half, inserted into the tunnel bed, and fixed with a horizontal mattress suture. After that, healing abutment was performed; simple interrupted sutures were applied (Ethicon W8005, Johnson & Johnson) (Figure 8).

Postoperative recommendations included rinsing with 0.2% chlorhexidine gluconate mouthwash solution (Corsodyl, GlaxoSmithKline) twice a day for a week and receiving antibiotic (1 g Amoxiclavi, LEK, d.d., Slovenia) twice a day for 5 days. To relieve pain, 100 mg of Nimesulide (Nise; Dr. Reddy's Laboratories Ltd., India) was prescribed. We also recommended to minimize trauma at the site; no special diet was recommended. The sutures were removed 14 days after surgical treatment.

At 2 months after surgery, sufficient tissue volume in the vestibulooral direction was determined. The patient was referred for prosthetics.

### 2.3. Prosthetic Procedures

Prosthetic procedures were initiated after 2 months of healing. Visually pathological changes in the area of the installed implant were not determined; the condition and volume of the soft tissue were satisfactory. Additionally, no pathological changes were found on the contact periapical X-ray. The implant stability quotient (ISQ) value was 78.

Considering that tooth 11 restoration was part of a complex rehabilitation, the stage of prosthetics was performed simultaneously on the upper and lower jaws. At the first stage, a temporary crown was made and fixed on the implant, which was corrected 1 time in 2 weeks during 2 months, and then, we proceeded to permanent prosthetics. The impressions were taken with polyvinyl siloxane impression material (Impregum, 3M ESPE, St. Paul, MN, USA).

**Figure 9:**
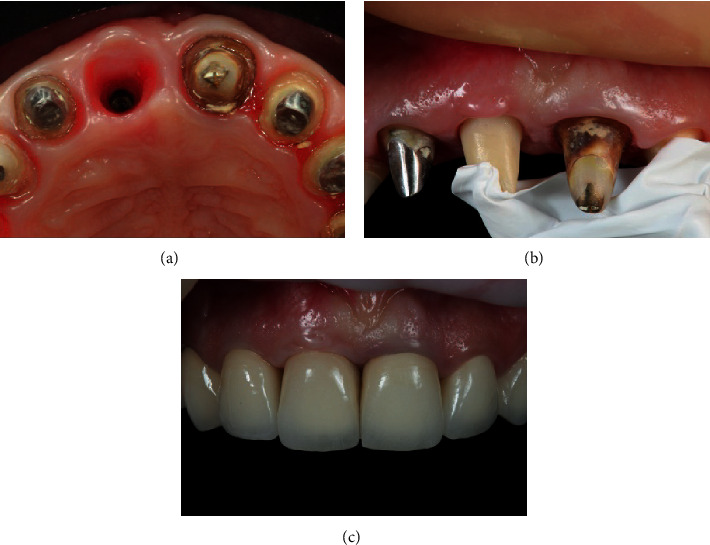
(a) Condition of soft tissues at the beginning of the prostheses stage. (b) Fixation of the individual zirconium abutment. (c) Final restoration.

The individual abutment of the implant was made of zirconium dioxide on a titanium base (Astra Tech-Dentsply Sirona, York, Pennsylvania). Then, the abutment was inserted into the titanium base. Accordingly, each part of the restoration was bonded to the titanium base in the following ways: both surfaces were treated with 50 microns of aluminium oxide particles at 2 bar pressure (0.25 MPa) for 20 s at a distance of 10 mm (RONDOflex Plus 360, KaVo, Germany). After that, a universal single-component priming agent (Monobond Plus, Ivoclar Vivadent, Liechtenstein) was applied to the zirconia and titanium bases accordingly. Dual-curing luting composite for the aesthetic and permanent cementation of ceramic (Variolink Esthetic DC, Ivoclar Vivadent, Liechtenstein) was used to lute the two components extraorally according to the manufacturer's recommendations. Abutment was fixed with torque 25 N/cm. The screw access hole was filled with temporary material. The final Е-Max (IVOCLAR Vivadent, Liechtenstein) restoration was fixed on Relyx U200 cement (3M ESPE, St. Paul, MN, USA) using a rubber dam (Figure 9).

Postoperative instructions including hygiene care were advised to the patient.

**Figure 10:**
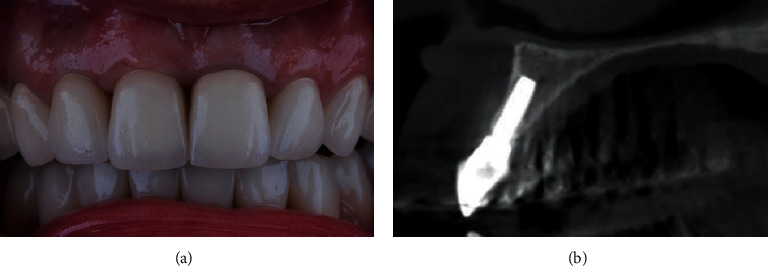
(a) 7 years after the treatment. (b) Stable volume of bone tissue.

The multidisciplinary regular check-up was emphasized. The patient was followed at 6 months, 1-7 years postloading. A CBCT scan was taken after 7 years, and no any bone resorption was found around the implant platform. The soft tissues were completely healthy at the follow-up time (Figure 10).

## 3. Discussion

The restoration of a single implant in the anterior maxillary region is a very complicated and challenging task due to the aesthetic aspect. In addition to the correct position of the implant, the success of the treatment also depends on the quality and quantity of hard and soft tissues [12], especially important to consider the changes that occur in the post extracted socket, which was described by many authors [3, 12–14]. When simultaneous implantation is not possible, the contemporary approach implies carrying out the method of saving the parameters of the socket [15]. For example, a systematic review and meta-analysis by Bassir et al. in 2018 demonstrated that using alveolar ridge preservation techniques after tooth extraction is effective in minimizing loss of horizontal and vertical hard tissue volume. No clear advantages have yet been identified in using bone substitute material or barrier membranes for this procedure [5]. Despite this fact, most scientists conclude on the feasibility of this procedure because it gives possibilities to reduce bone resorption and achieve better conditions for further implant treatment [4, 16–18]. In our clinical case, socket augmentation also allowed us to obtain a sufficient volume of bone for subsequent implant placement, while using a quickly resolvable bone-grafting material Bio-OSS collagen made it possible to carry out implant placement 4 months later.

Despite successful preservation of bone tissue parameters, vestibular collapse of soft tissues was revealed at the time of implant placement. In practice, according to a number of authors, the procedure of saving the socket parameter allows us to save most of the bone volume [19]. Most of the authors suggest combining socket preservation techniques with soft tissue augmentation, which helps to increase soft tissue volume and thickness and allows to prevent a vestibular collapse [7, 12]. The gold standard for this procedure is the use of an autogenous connective tissue graft. However, recently, there have been more and more reports of successful use of soft tissue substitute materials [7, 20]. In our clinical case, we used a free connective tissue graft from the maxilla tuber, which was fixed to the vestibular covering flap.

The healing process was auspicious, and the clinical picture in the early postoperative period was good. Although, 3 months later, soft tissue collapse was detected on the vestibular surface.

This clinical situation could be caused by many factors such as multiple alterations in the alveolar ridge following tooth extraction, partial resorption of the vestibular bone due to mucoperiosteal flap elevation, and connective tissue structure of the donor area.

The last factor is improbable, as the thickness of connective tissue in the tuber area is significantly greater compared to the hard palate, while the maintenance of adipose tissue and glandular tissue is minimal according to current data [21].

Nevertheless, using deepithelialized free connective tissue graft at the second stage of the implant surgery procedure and the healing abutment placement allowed us to reach a stable state of the contour of the alveolar ridge by the time of prosthetics and even after 7 years after complete treatment.

This clinical report demonstrated what changes in parameters of bone are possible after tooth removal and preservation of the socket. The reasons that caused soft tissue collapse after soft tissue augmentation, as well as the question of which stage of treatment is more suitable for soft tissue augmentation in similar cases, remain the subjects of discussion. However, it was concluded that an additional surgery to augment the soft tissues around dental implants may be a good solution for such clinical situations as the one presented.

## 4. Conclusion

The aim of the present study was to assess the possibilities of socket preservation technique and soft tissue augmentation in the anterior maxillary region. This case showed that these procedures are effective enough to preserve the parameters of the socket after tooth removal, and soft tissue augmentation allows to achieve satisfactory aesthetic outcomes. However, during the rehabilitation process, difficulties occur that clinicians may encounter and that need to be taken into account. For example, it may be the need for additional surgical intervention to achieve long-term stable condition of the soft tissues around dental implants. The scientific significance of this case report is that it has been demonstrated the necessity of further investigations of factors that may affect the success of soft tissue augmentation in the frontal teeth area during implant treatment, in particular when combining a soft tissue augmentation procedure with socket preservation. This study can expand existing knowledge on this topic and help clinicians to achieve favorable results while taking into account the experience that was described.

## Abbreviations

CBCT: Cone-beam computed tomography
CTG: Connective tissue graft
ISQ: Implant stability quotient.
